# The Impact of Successful Cataract Surgery on Quality of Life, Household Income and Social Status in South India

**DOI:** 10.1371/journal.pone.0044268

**Published:** 2012-08-31

**Authors:** Robert P. Finger, David G. Kupitz, Eva Fenwick, Bharath Balasubramaniam, Ramanathan V. Ramani, Frank G. Holz, Clare E. Gilbert

**Affiliations:** 1 Department of Ophthalmology, University of Bonn, Bonn, Germany; 2 Centre for Eye Research Australia, Royal Victorian Eye and Ear Hospital, University of Melbourne, Melbourne, Australia; 3 Sankara Eye Care Institutions, Coimbatore, India; 4 International Centre for Eye Health, London School for Hygiene and Tropical Medicine, London, United Kingdom; California Pacific Medicial Center Research Institute, United States of America

## Abstract

**Background:**

To explore the hypothesis that sight restoring cataract surgery provided to impoverished rural communities will improve not only visual acuity and vision-related quality of life (VRQoL) but also poverty and social status.

**Methods:**

Participants were recruited at outreach camps in Tamil Nadu, South India, and underwent free routine manual small incision cataract surgery (SICS) with intra-ocular lens (IOL) implantation, and were followed up one year later. Poverty was measured as monthly household income, being engaged in income generating activities and number of working household members. Social status was measured as rates of re-marriage amongst widowed participants. VRQoL was measured using the IND-VFQ-33. Associations were explored using logistic regression (SPSS 19).

**Results:**

Of the 294 participants, mean age ± standard deviation (SD) 60±8 years, 54% men, only 11% remained vision impaired at follow up (67% at baseline; p<0.001). At one year, more participants were engaged in income generating activities (44.7% to 77.7%; p<0.001) and the proportion of households with a monthly income <1000 Rps. decreased from 50.5% to 20.5% (p<0.05). Overall VRQoL improved (p<0.001). Participants who had successful cataract surgery were less likely to remain in the lower categories of monthly household income (OR 0.05–0.22; p<0.02) and more likely to be engaged in income earning activities one year after surgery (OR 3.28; p = 0.006). Participants widowed at baseline who had successful cataract surgery were less likely to remain widowed at one year (OR 0.02; p = 0.008).

**Conclusion:**

These findings indicate the broad positive impact of sight restoring cataract surgery on the recipients’ as well as their families’ lives. Providing free high quality cataract surgery to marginalized rural communities will not only alleviate avoidable blindness but also - to some extent - poverty in the long run.

## Introduction

Globally, cataract is the main cause of blindness with the vast majority of cataract blind living in low income countries (LIC) [1] and approximately eight million of those blind from cataract live in India. [2], [3] Blindness and poverty are closely linked in a cyclic relationship, as poverty can lead to blindness from conditions such as cataract or trachoma, and blindness can worsen poverty through reduced economic productivity. [4], [5], [6], [7], [8] Furthermore visual impairment leads to reduced quality of life (QoL) [9], [10], poorer general health [11], lower social status and increased mortality. [12], [13] Cataract surgery is a highly cost effective intervention [14] and cataract surgical rates have increased considerably over the past decade in many LICs, including India. However, the quality of provided cataract surgery is not always optimal, with a considerable proportion of patients still blind or vision impaired after cataract surgery in a LIC setting. [15], [16] Restoration of vision, for example through cataract surgery, has been demonstrated to enhance quality of life and participation in daily living and, more recently, to improve household economic status. [4].

The Millennium Development Goals (MDGs) are eight international development goals which the United Nations (UN) and all its member states aim to achieve by the year 2015. They include eradicating extreme poverty, gender equality, and developing a global partnership for development. [17] With its high success rates and cost-effectiveness, it is likely that the provision of cataract surgery may contribute towards reducing poverty as part of the MDGs.

Against this background, we assessed the impact of successful first eye cataract surgery on poverty, social status and vision-related QoL (VRQoL) in South India. We hypothesized that successful cataract surgery would reduce poverty, and improve VRQoL and social status. This information is important for the MDGs to achieve their goals and it may also help in allocating resources to ascertain high quality cataract surgical outcomes.

## Methods

### Ethics Statement

Ethical approval was obtained from the ethics committees of Sankara Eye Care Services and the University of Bonn. The study adhered to the tenets of the declaration of Helsinki. Every participant gave informed, written (signature or thumb imprint) consent.

This prospective study, took place from March 2009 until July 2010 in Tamil Nadu, India. Tamil Nadu is an industrialized and populous state (population 62 million in 2001) with the largest urban conglomeration in India. [18] However, there are still poor, rural areas, which are underserviced as providers tend to be concentrated in cities. The cataract surgical rate in Tamil Nadu is above the Indian average, being approximately 4000 cataract operations per million population per year. [19] This study was embedded within routine services provided by one community eyecare provider, Sankara Eye Care Services, Coimbatore. The cataract outreach program operated by Sankara has been described in detail elsewhere. [20].

### Recruitment and Follow Up

Participants who were visually impaired from cataract and had not undergone prior cataract surgery in any eye were eligible. Persons who were classified as poor, aged 40 years or more, and eligible for first eye cataract surgery were recruited. Visual impairment was defined as less than 6/60 (logarithm of minimum angel of resolution (LogMAR) 1.0) in the eye assigned for surgery.

Recruitment took place at the base hospital after participants had been assessed at outreach eye clinics, which are regularly conducted. Those who agreed to participate were interviewed and underwent a full eye examination before undergoing cataract surgery. Patients’ transport, surgery, and inpatient hospital stays were provided free by the hospital as patients all fell under the poverty threshold (defined below). After cataract surgery, patients were given eye drops, transported back to the outreach site and told to attend the next outreach clinic (usually a month later) for a follow up assessment of the cataract surgery. If they failed to attend they were sent a reminder by mail. Follow-up data collection for the study occurred at the patients’ homes at 1 year follow up by community eye health workers local to the area who are employed full-time by Sankara.

### Ocular Assessment and Cataract Surgery

Preoperative assessment included distance visual acuity (DVA) measurement using a LogMAR numbers or tumbling E chart at six meters without correction or with habitual correction and with pinhole. Participants underwent a basic eye examination by an ophthalmologist to determine the cause of visual loss and underwent a more detailed eye examination, including pupil dilation, if the cause of visual loss could not be determined. Only participants for whom cataract was the main cause of visual impairment were included. At follow up, DVA was tested using the same chart at six meters with habitual correction or uncorrected and with pinhole.

Manual small incision cataract surgery (SICS) with implantation of an intraocular lens (IOL) (rigid, single piece PMMA implant) under parabulbar anaesthesia was performed on all participants. IOL power was determined for each individual using manual keratometry (Bausch and Lomb) and an ocular ultrasound A scan (Echorule 2, Biomedix Optotechnik & Devices, India).

### Measures of Poverty

Participatory approaches in ranking wealth have been found to yield useful data and valid information on household wealth. [21], [22] The economic part of the baseline questionnaires was extensively discussed with key informants and patients in focus groups prior to the current study in order to reflect meaningful and culturally appropriate measures of poverty. Household income is commonly considered a gold standard measure of current socioeconomic position. [22] The most commonly used definition of global poverty is the absolute poverty line set by the World Bank, based on income and/or consumption (poverty $2 and extreme poverty $1 a day or less). [23] The Indian government defined poverty as less than Indian Rupees (Rs). 560 per month in urban areas and Rs. 368 in rural areas in 2006 [24] although definitions used by state governments may vary. In this study, poverty was defined as access to less than Rs. 1200 a month, either as personal (sole earner) or household income. This threshold is in agreement with Tamil Nadu state policy where governmental ration cards are issued when the monthly household income is less than Rs. 1200 (approx. US$25).

In the current study poverty was measured through self-reported monthly household income, employment status, occupation and number of working household members. As asset ownership was unlikely to change over the 1-year follow up period and could thus not reflect the impact of successful first eye cataract surgery, it was not assessed in this study. [5] During the baseline interview, participants were encouraged to discuss monthly household income with accompanying household members and include all sources of income, monetary and non-monetary into the final estimate. As blind persons have been found to be more likely to be unemployed and, if employed, to work more often in low wage jobs [13], we recorded employment status and occupation of all participants at baseline and follow up. In addition, the number of working household members was recorded, as households with disabled members generally have less members involved in income generating activities of any kind. [13] Working was defined as being involved in activities which directly or indirectly generate income.

### Measures of Social Status

Disability, including blindness, leads to social exclusion and stigmatization which in turn impedes access to social networks and formal services or social institutions. [13] Widowers and even more so widows are particularly affected by this societal response in India, and have little resources at their disposal to cope with disability. [13] More disabled women than men are unmarried or do not remarry once widowed in India. [25] As social status or stigmatization is difficult to measure [26], we assessed whether participants who were widowed at baseline remarried following cataract surgery as a proxy of decreasing stigma and increasing societal esteem as well as an improved financial outlook of the individual or household.

### Vision-related Quality of Life

VRQoL was measured using the IND-VFQ-33, a structured questionnaire which contains 33 questions (items) related to the degree of difficulty in performing vision-dependent activities (e.g., reading, climbing stairs), psychosocial impact (e.g. fear, anxiety) and visual symptoms (e.g. glare, pain). [27], [28] The original IND-VFQ-33 questionnaire was developed and extensively validated in the same Indian state (Tamil Nadu), thus no cultural or linguistic adaptation was necessary. [27] In the current study, we performed Rasch analysis to assess the measurement properties of the IND-VFQ-33 in our sample population over time.

### Sample Size Calculation

As no data were available to estimate the effect of cataract surgery on household income or the rate of re-marriage, we based the sample size calculations on reported changes in per capita expenditure (PCE) following cataract surgery. [5] The observed change was of a similar magnitude in all cases regardless of whether patients were blind, severely or moderately visually impaired at baseline. Assuming an odds ratio of 1.6 for an improvement in categories of household income, with a power of 0.8 at a significance level of p = 0.05, we would need 293 participants (G*Power 3 [29]). Accounting for a loss of 10% to follow up, we aimed to recruit 330 participants.

### Psychometric Evaluation of the IND-VFQ-33

We have reported the process of psychometric evaluation of the IND-VFQ-33 using Rasch analysis in more detail elsewhere. [10] In brief, Rasch analysis is a modern psychometric method that mathematically describes the interaction between respondents and test items. We performed Rasch analysis using Winsteps software (version 3.68), Chicago, Illinois, USA. [30] It was important to establish that differences between the IND-VFQ scores at baseline and follow-up are valid indicators of changes over time. [31] Consequently, the baseline and follow-up data were stacked and the absence of differential item functioning (DIF) was used to establish invariance over time. Any change in VRQoL scores on an individual level was considered clinically meaningful if it was larger than approximately half the standard deviation of the overall mean. This is generally considered to be a useful estimate of a clinically meaningful difference [32], [33], and has repeatedly been used to rate the meaningfulness of change in parameters such as VRQoL or vision-specific functioning. [10].

### Statistical Analysis

The SPSS statistical software (Version 19.0, SPSS Science, Chicago, IL) was used to analyze the data. Participants lost to follow up were excluded from all analyses. Descriptive statistical analyses were performed to characterize the participants’ sociodemographic, clinical and IND-VFQ-33 data. Logistic regression (binary and multinomial) models were conducted to determine the independent factors associated with measures of poverty, social status and VRQoL.

Visual acuity was converted into LogMAR for analysis. Successful cataract surgery was defined as a VA improvement equal or better than 20/63 (LogMAR 0.5). In order to demonstrate the impact of cataract surgery, analyses were based on successful cataract surgery, rather than the presence or absence of vision impairment at follow-up, as these two variables contain the same information. In 80% of cases the operated eye was the better eye at follow up.

## Results

### Socio-demographics and Clinical Characteristics of the Participants

A total of 313 individuals were recruited at baseline. 19 (6%) patients were lost to follow up and a further 21 (7%) participants who underwent second eye cataract surgery during follow up were excluded from all but the descriptive analyses. Baseline characteristics of patients lost to follow up were not significantly different (p>0.05) with regards to age, gender, better eye VA, household size or income. The final study sample at baseline thus comprised 294 participants with a mean ± SD age of 60±8 years. Just under half of the participants were women (46%, **Table 1**). Vision significantly improved after cataract surgery (better eye LogMAR 0.7 (baseline) to 0.3 (follow up), p<0.001), and only 11% of patients remained vision impaired compared to 67% at baseline, p<0.001.

**Table 1:** Characteristics of the sample at baseline and 1 year follow up.

		Baseline	1 year Follow up	p-value
		n = 294	n = 294	BL-1 yr
Age in yrs		60.07±8.29		
Gender	Female	134(45.6%)		
Education	No schooling	125(42.5%)		
	Up to 5 yrs	100(34.0%)		
	More than 5 yrs	54(18.4%)		
**Mean** ± **standard deviation**				
Visual acuity in the better eye		0.74±0.44	0.27±0.20	<0.001
VA of operated eye+		1.29±0.65	0.34±0.29	<0.001
VRQoL – subscales of the IND-VFQ 33, logits				
• Activity Limitation		65±24	80±9	<0.001
• Mobility		51±23	70±7	<0.001
• Psychosocial Impact		59±21	79±13	<0.001
No. of household members		3.87±2.17	4.00±1.99	.100
Working household members		0.97±0.75	1.49±0.70	<0.001
**Proportion n(%)**				
Marital status	Married	208(70.7%)	232(78.9%)	<0.001
	Widowed or Single	80(27.2%)	49(16.7%)	
Visual Impairment		196 (66.7%)	32 (10.9%)	<0.001
Working		128(43.5%)	225(76.5%)	<0.001
Occupation$	Unskilled labour (daily wage)	86(43.8%)	177(78.7%)	.061
	Land owner	16(12.5%)	32(14.2%)	
	Skilled labour	26(20.3%)	16(7.1%)	
Reason for not working^#^	Vision problem	76(45.8%)	4(5.8%)	.061
	Too old or retired	32(19.3%)	33(47.8%)	
	Other health problem	29(17.5%)	19(27.5%)	
	Doesn’t need to work	21(12.7%)	7(10.1%)	
Household’s monthly income	Rs 0–1000	143(48.7%)	59(20.1%)	<0.001
	Rs 1001–3000	92(31.3%)	207(70.4%)	
	>Rs 3000	28(9.5%)	20(6.8%)	

BL = baseline, VA = visual acuity, measured in LogMAR, VRQoL = vision-related quality of life, RS = Indian Rupees;

+ Operated eye VA at baseline was taken before cataract surgery;

$ question applicable to working participants only;

#question applicable to the non working participants only; p-values derived from paired sample t-tests for continuous and Wilcoxon signed rank test for categorical variables.

### Measures of Poverty

Monthly household income increased to at least the next better category in 122 persons (45.5%) with most participants reporting to be in the Rs 1001–3000 category at follow up (**Table 1**, p<0.001). The mean number of working household members significantly increased at follow up (0.97 to 1.49, p<0.001, **Table 1**). Similarly, the number of participants engaged in income generating activities increased from 128 (44%) at baseline to 225 (77%, p<0.001) at follow up, with the largest increase seen in the unskilled, daily wage category (**Table 1**). The number of participants reported to not work due to vision problems decreased from 76 at baseline to 4 at follow up.

Participants who had successful cataract surgery were significantly more likely to report a higher monthly household income 1 year after cataract surgery. Compared to the highest income category (>3000 Rs./month), participants were about five times (OR 0.22, 95%CI 0.08–0.62; p = 0.004) less likely to report a monthly household income of 0–1000 Rs. and about twenty times less likely to report an income of >1000–3000 Rs. (OR 0.05; 95%CI <0.01–0.64; p = 0.021, **Table 2**). Participants who had successful cataract surgery were more likely to be engaged in income earning activities one year after surgery (OR 3.28; 95% CI 1.40–7.82; p = 0.006, **Table 2**).

**Table 2:** Impact of successful cataract surgery on marital status and measures of poverty at follow up.

		OR	95% CI	p
**Marital (social) status**	Widowed	1	reference	
	Married	3.28	1.31; 8.23	**0.012**
**Working/Occupation**	No	1	reference	
	Yes	3.31	1.40; 7.82	**0.006**
**No. of working** **household members**		1.24	0.55; 2.81	0.603
**Monthly household income**	> Rs 3000	1	reference	
	>1000–3000	0.05	<0.01; 0.64	**0.021**
	0–1000	0.22	0.08; 0.62	**0.004**

OR = odds ratio, CI = confidence interval; controlling for age, gender, education and household size (no. of members) in logistic regression.

### Social Status

Participants who had successful cataract surgery were less likely to remain widowed at one year (OR 0.02; 95% CI <0.01–0.35; p = 0.008, **Table 3**). At baseline, 208 (71%) participants were married, and 80 (27%) widowed or single (**Table 1**). Over the course of the year, 28 participants remarried, while 47 participants remained widowed (5 lost to follow up, **Table 3**). Stratifying this by gender, nine out of 13 widowers (69%) and 19 out of 62 widows (31%) remarried over the course of the study.

**Table 3:** Characteristics of participants who re-married and participants who were still widowed/single at 1 year (5 participants lost to follow up).

		Univariate analysis			Logistic regression		
		Remarried n = 28	Still widowed n = 47	p	OR	95% CI	p
Age		59.93±6.95	59.04±8.24	0.635	0.98	0.90; 1.07	0.696
Gender	Male	9(32.1%)	4(8.5%)	**0.009**	0.10	0.01; 1.17	0.066
	Female	19(67.9%)	43(91.5%)		1	(reference)	
Education	No schooling	16(57.1%)	37(78.7%)	**0.035**	25.51	0.89; 728.45	0.058
	Up to 5 yrs	7(25.0%)	8(17.0%)		15.92	0.57; 444.82	0.103
	More than 5 yrs	4(14.3%)	1(2.1%)		1	(reference)	
VRQoL:							
• Mobility		71±7	70±8	0.779	Not included		
• Activity Limitation		80±9	80±9	0.567	Not included		
• Psychosocial Impact		82±13	79±12	0.322	Not included		
VI at BL		18(64.3%)	32(68.1%)	0.737	Not included		
VI at 1 year		2(7.1%)	10(21.3%)	0.109	Not included		
Successful cataract surgery at 1 year		25(89.3%)	32(68.1%)	**0.039**	0.02	<0.01; 0.35	**0.008**
Working at 1 yr		23(82.1%)	33(70.2%)	0.254	0.95	0.17; 5.19	0.952
Monthly household income at 1 yr	Rs 0–1000	8(28.6%)	15(31.9%)	0.670	0.11	<0.01; 8.14	0.310
	Rs 1001–3000	19(67.9%)	30(63.8%)		0.53	0.01; 22.99	0.743
	>Rs 3000	1(3.6%)	1(2.1%)		1	(reference)	
No. of household members at 1 yr		3.79±1.79	3.68±2.17	0.830	1.07	0.67; 1.71	0.782
No. of working household members at 1 yr		1.44±75	1.36±.74	0.626	0.59	0.14; 2.41	0.459

Data reported as mean ± standard deviation (SD) or n(%); p-values for univariate tests derived from independent samples t-tests for continuous and Mann-Whitney U test for categorical variables; OR = odds ratio; CI = confidence interval.

### Vision-related Quality of Life

The psychometric properties of the IND-VFQ-33 are summarized in **table S1**. The IND-VFQ-33 was split into four subscales, mobility, activity limitations, psychosocial impact and visual symptoms. All subscales fit the Rasch model (table S1). However, the visual symptoms subscale was left out of all further analyses as it was not felt to add any essential information. All other subscales demonstrated an improvement in VRQoL after cataract surgery (all p≤0.001, **Table 1**). Having had successful cataract surgery was independently associated with higher reported mobility, less activity limitations and better psychosocial impact (all p<0.05; **Table 4**). Marital or work status was not associated with any of the subscale scores. Participants reported better emotional well-being with an improvement of monthly household income at one year (OR 31, p = 0.034, **Table 4**).

**Table 4:** Factors associated with patient-reported quality of life at 1 year in generalized linear models, adjusted for age, gender and education.

		Mobility			Activity Limitation			Psychosocial Impact		
		OR	95% CI	p	OR	95% CI	p	OR	95% CI	p
**Successful cataract surgery**	No (ref)	1			1			1		
	Yes	1375.50	127.17; 14877.60	**<0.001**	30.43	1.51; 611.92	**0.026**	5033.27	62.86; 403027.84	**<0.001**
**Working**	No(ref)	1			1			1		
	Yes	1.19	0.12; 11.99	0.884	4.15	0.22; 76.49	0.339	1.44	0.02;101.99	0.866
**Marital status**	Widowed/Single (ref)	1			1			1		
	Married	0.16	0.01; 2.32	0.179	0.34	0.01; 9.91	0.533	0.20	<0.01;27.78	0.526
**Monthly household income**	No improvement(ref)	1			1			1		
	Improvement	1.34	0.24; 7.52	0.740	1.76	0.20; 15.49	0.611	31.32	1.31;751.00	**0.034**

OR = odds ratio, CI = confidence interval, ref =  reference category.

## Discussion

Persons who underwent successful cataract surgery reported better visual acuity and increased VRQoL in South India. Successful cataract surgery also increased the likelihood to be engaged in an income earning activity, report a higher monthly household income and report a higher number of working household members one year on. In addition, widowed or single participants who had successful cataract surgery were more likely to have remarried over the one year follow up period. These findings emphasize the need for high quality cataract surgery services, as unsuccessful cataract surgery may not only lead to no improvements in vision and VRQoL, but may also deprive patients of a possible future reduction of poverty at the household level and their chance to re-marry in case of widowhood.

Overall, our findings are in line with other studies, where blindness, in particular from cataract, and poverty have been found to be intricately linked. [4], [6], [7], [34] However, there is a dearth of literature regarding the non-ocular impact of cataract surgery in low income countries. [5] Previous studies have reported that cataract surgery may lead to an improvement in VRQoL [35], per capita expenditure [5] and an increase in time spent on productive activities. [36] These non-ocular outcomes of cataract surgery are reflected by our findings of an increase in VRQoL, monthly household income, the number of working household members and the likelihood to be engaged in income generating activities one year after cataract surgery.

Several studies have found poverty to be a barrier to accessing cataract surgery services in India and elsewhere. [37], [38], [39] In addition, poor surgery outcomes are very likely to discourage acceptance of available cataract surgery services. [7], [20] Given the important positive outcomes of cataract surgery found in this study, increased efforts are needed to encourage greater acceptance of offered cataract surgery services. Such services should ideally be of high quality and provided regularly by the same provider in the same vicinity, and tailored to the needs of impoverished communities. [20].

The authors are unaware of any other study which has accessed the impact of a vision-restoring intervention on social status in blind persons in South Asia. In our study, successful cataract surgery, i.e. sight restoration to levels above vision impairment, was associated with an increased likelihood of being remarried a year later if widowed at baseline. Based on published studies, one can assume a rate of remarriage after being widowed of 6–20% for women and 60–65% for men of all ages over their remaining life span in South India. [40] Unfortunately, no reports of how an existing disability affects rates of remarriage in India are available. Thus, reported rates only reflect rates of remarriage in the general population. In our sample, these high rates of re-marriage were observed in an older population during a one-year follow-up, which increases the probability of the observed rates being higher than in the general population. Whether the positive economic impact of successful cataract surgery highlighted above increases the likelihood of remarriage, or whether it is reduced stigma due to sight restoration, or a combination of these, is difficult to assess. In either case, our results suggest that successful cataract surgery may increase the likelihood of widowed persons remarrying. However, as our observations are based on a small sample and a limited follow-up with no control group, they have to be interpreted with caution. Nevertheless, this finding has a range of positive implications, as widowhood is associated with adverse health impacts, loss of opportunities to engage in income generating activities and loss of societal esteem. [41].

Strengths of our study include the provision of uniform cataract surgery of high quality with IOL implantation, with good surgical outcomes, detailed visual acuity data, little attrition to follow up and culturally appropriate and well validated questionnaires. Moreover, being embedded into routine service provision of Sankara Eye Care Services in Coimbatore, our sample is representative of the communities served by this service provider in Tamil Nadu, South India. Economic data were collected at the individual and household level, rather than inferred from district or other regional approximations such as postcodes or census data which increases accuracy. [6], [42] During the one year follow-up, no new government or NGO funded programs increasing options to be engaged in income generating activities were implemented in the area. To the authors’ knowledge, no new factory was opened or other large employer moved into the area, either. The use of Rasch analysis, an important step in modern scale validation, to assess the measurement properties of the IND-VFQ-33 is another strength of this study. [10] Moreover, as the IND-VFQ-33 was developed using input primarily from cataract patients in Tamil Nadu, [28] the item content is likely to be very appropriate for this sample.

Conversely, our study is limited by a relatively small sample size, and a relatively short follow up to assess the long term impact of cataract surgery on VRQoL, poverty and social status. The lack of a non-operated control group makes it difficult to generalize results. However, other case-control studies have demonstrated the overall impact of cataract surgery compared to no surgery. [4] Also, our measures of poverty differ from other studies assessing the association of blindness and poverty, which limits comparability of our findings. Assessing the impact of successful cataract surgery on rates of re-marriage is inherently difficult and our results have to be interpreted with caution. However, overall, our results compare well to other studies as well as studies assessing rates of re-marriage based on Indian census data. [40].

In conclusion, successful cataract surgery restores not only vision and improves VRQoL, but enables previously visually impaired persons to restart work, leads to a higher monthly household income, and more members of the household being engaged in income earning activities. In addition, it makes re-marriage amongst widowed elderly persons more likely. Thus, it is a public health imperative to provide high quality cataract surgery to impoverished communities in developing countries as part of achieving the MDGs.

## Supporting Information

Table S1 **The fit parameters of the altered IND-VFQ 33 compared to the Rasch model.**
(DOCX)

## References

[1] Pascolini D, Mariotti SP (2012) Global estimates of visual impairment: 2010. British J Ophthalmol 96: 614–618. 10.1136/bjophthalmol-2011-300539 22133988

[2] Murthy G, Gupta SK, John N, Vashist P (2008) Current status of cataract blindness and Vision 2020: the right to sight initiative in India. Indian J Ophthalmol 56: 489–494. 18974520 10.4103/0301-4738.42774 PMC2612994

[3] Finger RP (2007) Cataracts in India: current situation, access, and barriers to services over time. Ophthalmic Epidemiol 14: 112–118. 17613845 10.1080/09286580601114967

[4] Kuper H, Polack S, Eusebio C, Mathenge W, Wadud Z, et al (2008) A case-control study to assess the relationship between poverty and visual impairment from cataract in Kenya, the Philippines, and Bangladesh. PLoS Med 5: e244. 19090614 10.1371/journal.pmed.0050244 PMC2602716

[5] Kuper H, Polack S, Mathenge W, Eusebio C, Wadud Z, et al (2010) Does cataract surgery alleviate poverty? Evidence from a multi-centre intervention study conducted in Kenya, the Philippines and Bangladesh. PLoS One 5: e15431. 21085697 10.1371/journal.pone.0015431 PMC2976760

[6] Gilbert CE, Shah SP, Jadoon MZ, Bourne R, Dineen B, et al (2008) Poverty and blindness in Pakistan: results from the Pakistan national blindness and visual impairment survey. BMJ 336: 29–32. 18087076 10.1136/bmj.39395.500046.AE PMC2174750

[7] Dandona L, Dandona R, Srinivas M, Giridhar P, Vilas K, et al (2001) Blindness in the Indian state of Andhra Pradesh. Investigative Ophthalmology and Visual Science 42: 908–916. 11274066

[8] Zimmer Z (2008) Poverty, wealth inequality and health among older adults in rural Cambodia. Soc Sci Med 66: 57–71. 17913320 10.1016/j.socscimed.2007.08.032 PMC2171035

[9] Polack S, Eusebio C, Fletcher A, Foster A, Kuper H (2010) Visual impairment from cataract and health related quality of life: results from a case-control study in the Philippines. Ophthalmic Epidemiol 17: 152–159. 20455844 10.3109/09286581003731536

[10] Finger RP, Kupitz DG, Holz FG, Balasubramaniam B, Ramani RV, et al (2011) The impact of the severity of vision loss on vision-related quality of life in India: an evaluation of the IND-VFQ-33. Investigative Ophthalmology and Visual Science 52: 6081–6088. 21693607 10.1167/iovs.11-7388

[11] Taylor HR, Katala S, Munoz B, Turner V (1991) Increase in mortality associated with blindness in rural Africa. Bull World Health Organ 69: 335–338. 1893509 PMC2393108

[12] Sengupta M, Agree EM (2002) Gender and Disability Among Older Adults in North and South India: Differences Associated with Coresidence and Marriage. Journal of Cross-Cultural Gerontology 17: 313–336. 14617962 10.1023/a:1023079219538

[13] Foley D, Chowdhury J (2007) Poverty, Social Exclusion and the Politics of Disability: Care as a Social Good and the Expenditure of Social Capital in Chuadanga, Bangladesh. Social Policy & Administration 41: 372–385.

[14] Baltussen R, Sylla M, Mariotti SP (2004) Cost-effectiveness analysis of cataract surgery: a global and regional analysis. Bull World Health Organ 82: 338–345. 15298224 PMC2622839

[15] Dandona L (2001) Cataract surgery in very elderly patients. Outcome of cataract surgery is poor in developing countries. BMJ 323: 455. 11548732

[16] Dandona L, Dandona R, Anand R, Srinivas M, Rajashekar V (2003) Outcome and number of cataract surgeries in India: policy issues for blindness control. Clinical and Experimental Ophthalmology 31: 23–31. 12580890 10.1046/j.1442-9071.2003.00595.x

[17] United Nations (2012) United Nations Millenium development Goals. New York: United Nations.

[18] Office of the Registrar General and Census Commissioner (2001) Census of India. Census data 2001: India at a glace. Government of India.

[19] World Health Organization (2004) Cataract Surgical Rate Southeast Asia Region 2004. Geneva: World Health Organization.

[20] Finger RP, Kupitz DG, Holz FG, Chandrasekhar S, Balasubramaniam B, et al. (2011) Regular provision of outreach increases acceptance of cataract surgery in South India. Tropical Medicine & International Health. 10.1111/j.1365-3156.2011.02835.x 21718395

[21] Adams AM, Evans TG, Mohammed R, Farnsworth J (1997) Socioeconomic stratification by wealth ranking: Is it valid? World Dev 25: 1165–1172.

[22] Hargreaves JR, Morison LA, Gear JS, Kim JC, Makhubele MB, et al (2007) Assessing household wealth in health studies in developing countries: a comparison of participatory wealth ranking and survey techniques from rural South Africa. Emerg Themes Epidemiol 4: 4. 17543098 10.1186/1742-7622-4-4 PMC1894790

[23] World Bank (1990) World Development Report: Poverty. Washington: World Bank.

[24] Department of Planning (2006) Survey for below the poverty line families. Government of Punjab.

[25] Gooding K (2006) Poverty and Blindness: A survey of the literature. Haywards Heath: Sightsavers International Programme Development Unit. http://www.vision2020uk.org.uk/library.asp?section=000100050005.

[26] Krieger N, Williams DR, Moss NE (1997) Measuring social class in US public health research: concepts, methodologies, and guidelines. Annu Rev Public Health 18: 341–378. 9143723 10.1146/annurev.publhealth.18.1.341

[27] Gupta SK, Viswanath K, Thulasiraj RD, Murthy GV, Lamping DL, et al (2005) The development of the Indian vision function questionnaire: field testing and psychometric evaluation. Br J Ophthalmol 89: 621–627. 15834097 10.1136/bjo.2004.050732 PMC1772618

[28] Murthy GV, Gupta SK, Thulasiraj RD, Viswanath K, Donoghue EM, et al (2005) The development of the Indian vision function questionnaire: questionnaire content. Br J Ophthalmol 89: 498–503. 15774932 10.1136/bjo.2004.047217 PMC1772602

[29] Faul F, Erdfelder E, Lang AG, Buchner A (2007) G*Power 3: a flexible statistical power analysis program for the social, behavioral, and biomedical sciences. Behavior Research Methods 39: 175–191. 17695343 10.3758/bf03193146

[30] Linacre JM (2008) WINSTEPS Rasch measurement computer program. Chicago, IL: Winsteps.com.

[31] Wolfe EW, Chiu CW (1999) Measuring pretest-posttest change with a Rasch Rating Scale Model. J Outcome Meas 3: 134–161. 10204324

[32] Sloan J (2005) Assessing the minimally clinically significant difference: scientific considerations, challenges and solutions. COPD 2: 57–62. 17136963 10.1081/copd-200053374

[33] Norman G, Sloan J, Wyrwich K (2003) Interpreation of changes in health-related quality of life: the remarkable universality of half a standard deviation. Med Care 41: 582–592. 12719681 10.1097/01.MLR.0000062554.74615.4C

[34] Lewallen S (2008) Poverty and cataract–a deeper look at a complex issue. PLoS Med 5: e245. 19090615 10.1371/journal.pmed.0050245 PMC2602717

[35] Polack S, Eusebio C, Mathenge W, Wadud Z, Mamunur AK, et al (2010) The impact of cataract surgery on health related quality of life in Kenya, the Philippines, and Bangladesh. Ophthalmic Epidemiol 17: 387–399. 21090912 10.3109/09286586.2010.528136

[36] Polack S, Eusebio C, Mathenge W, Wadud Z, Rashid M, et al (2010) The impact of cataract surgery on activities and time-use: results from a longitudinal study in Kenya, Bangladesh and the Philippines. PLoS One 5: e10913. 20531957 10.1371/journal.pone.0010913 PMC2879361

[37] Nirmalan PK, Katz J, Robin AL, Krishnadas R, Ramakrishnan R, et al (2004) Utilization of eye care services in rural south India: the Aravind Comprehensive Eye Survey. British J Ophthalmol 88: 1237–1241. 10.1136/bjo.2004.042606 PMC1772350 15377541

[38] Kessy JP, Lewallen S (2007) Poverty as a barrier to accessing cataract surgery: a study from Tanzania. Br J Ophthalmol 91: 1114–1116. 17709583 10.1136/bjo.2006.112474 PMC1954924

[39] Finger RP, Ali M, Earnest J, Nirmalan PK (2007) Cataract surgery in Andhra Pradesh state, India: an investigation into uptake following outreach screening camps. Ophthalmic Epidemiol 14: 327–332. 18161605 10.1080/01658100701486814

[40] Bhat M, Kanbargi R (1984) Estimating the incidence of widow and widower re-marriages in India from census data. Population Studies 38: 89–103. 22087623 10.1080/00324728.1984.10412824

[41] Chen M, Dreze J (1992) Widows and health in rural north India. Economic and Political weekly 27: 24–31.

[42] Fraser S, Bunce C, Wormald R, Brunner E (2001) Deprivation and late presentation of glaucoma: case-control study. BMJ 322: 639–643. 11250847 10.1136/bmj.322.7287.639 PMC26542

