# Supplementary material for: The Impact of Successful Cataract Surgery on Quality of Life, Household Income and Social Status in South India
Source: PLoS One. 2012 Aug 31;7(8):e44268. doi: 10.1371/journal.pone.0044268 (PMC3432104; doi:10.1371/journal.pone.0044268)
Supplement: Table S1 — The fit parameters of the altered IND-VFQ 33 compared to the Rasch model. (DOCX) [file pone.0044268.s001.docx]

| **Supplemental Table 1.** The fit parameters of the altered IND-VFQ 33 compared to the Rasch model | | | | | |
| --- | --- | --- | --- | --- | --- |
| **Parameters** | **Rasch model** | **IND-VFQ 33 Rasch guided subscales** | | | |
|  |  | **Mobility** | **Activity Limitation** | **Psychosocial Impact** | **Visual Symptoms** |
| Item No. |  | 1-4, 7-8 | 10-14, 17-21 | 22-26 | 27-33 |
| No. of misfitting items | 0 | 0 | 0 | 0 | 0 |
| Person separation (PSI) | >2.0 | 2.83 | 2.90 | 2.12 | 2.29 |
| Person reliability (PR) | >0.8 | 0.89 | 0.89 | 0.82 | 0.84 |
| Person mean | 0 | 5.41 | 3.78 | 3.33 | 2.91 |
| Principal Components Analysis ( Eigenvalue for 1^st^ contrast) | <2.0 | 1.7 | 1.9 | 1.6 | 1.7 |
| Variance by the first factor | 50-60% | 64.5% | 65.1% | 65.5% | 65.2% |
